# Supplementary figures and images for: Maintenance of muscle mass in adult male mice is independent of testosterone
Source: PLoS One. 2021 Mar 25;16(3):e0240278. doi: 10.1371/journal.pone.0240278 (PMC7993603; doi:10.1371/journal.pone.0240278)

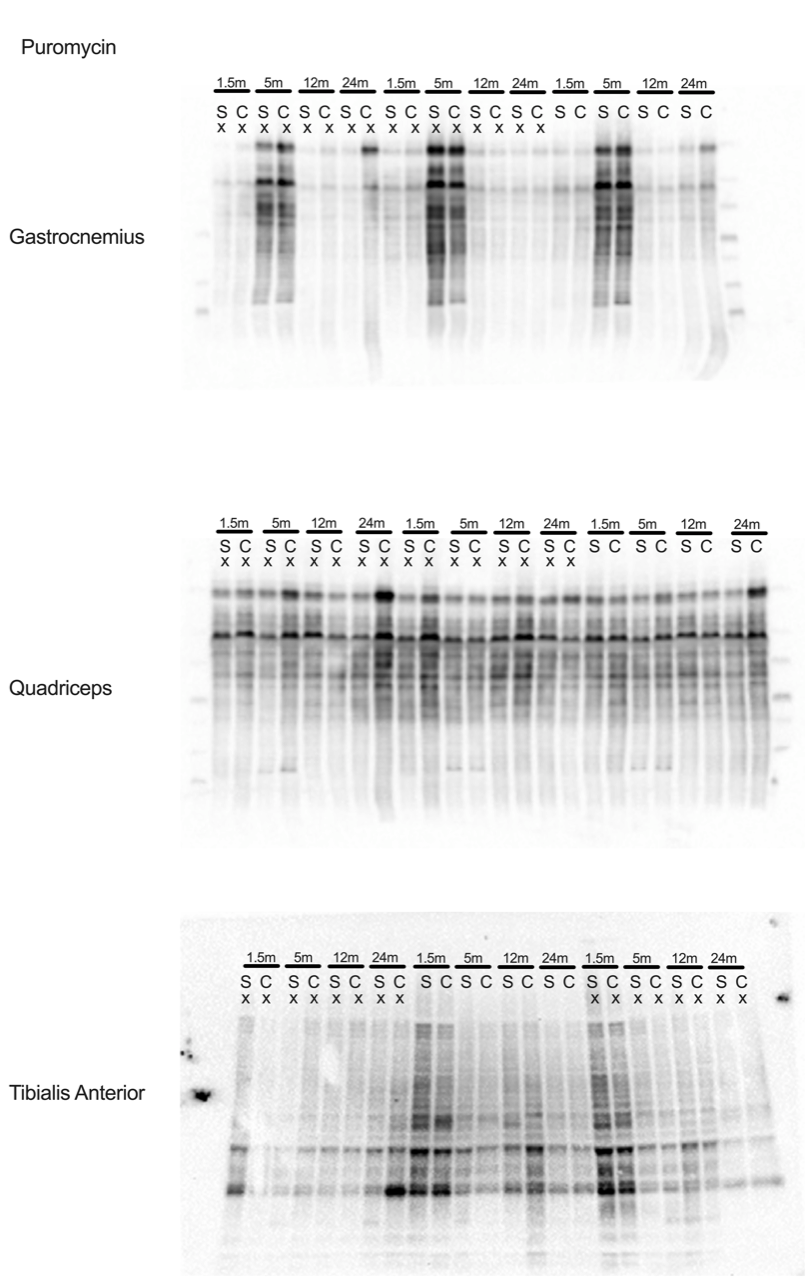

Supplement: S1 Fig — (TIFF) [file pone.0240278.s001.tiff]

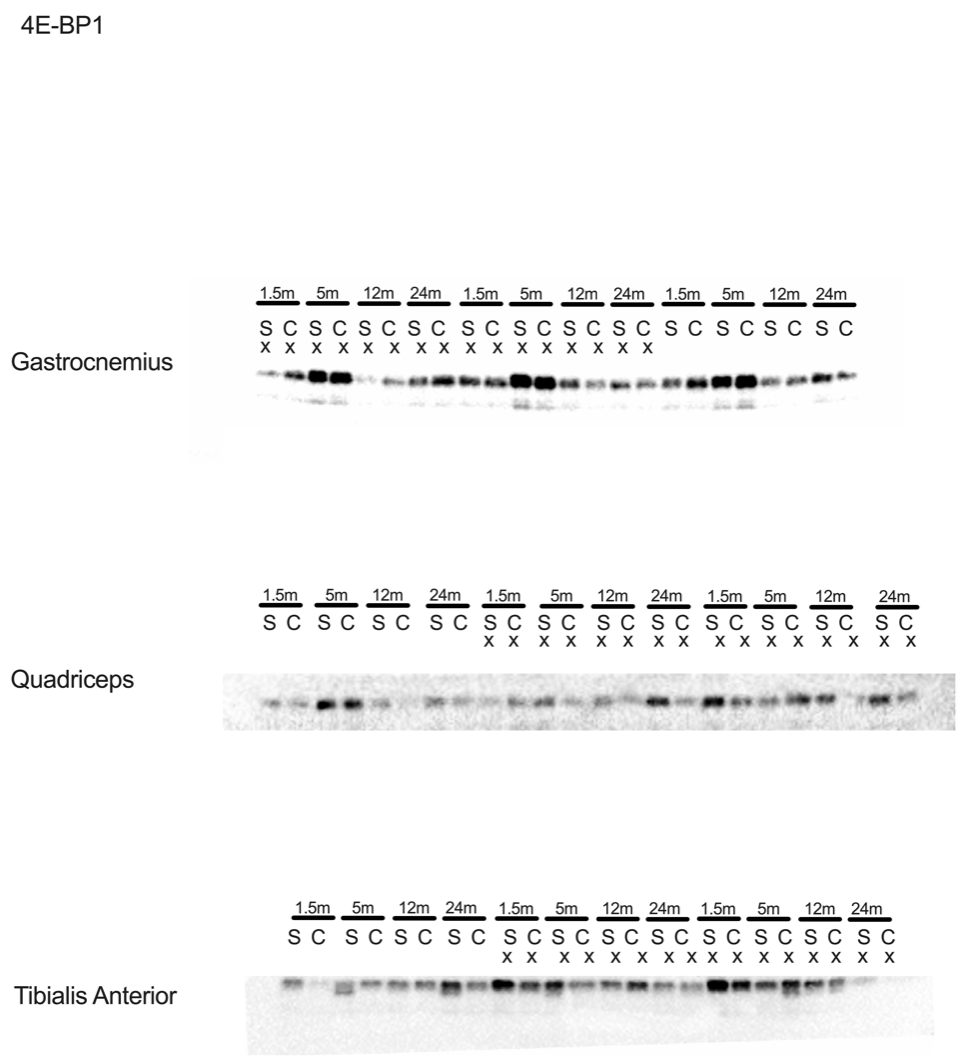

Supplement: S2 Fig — (TIFF) [file pone.0240278.s002.tiff]

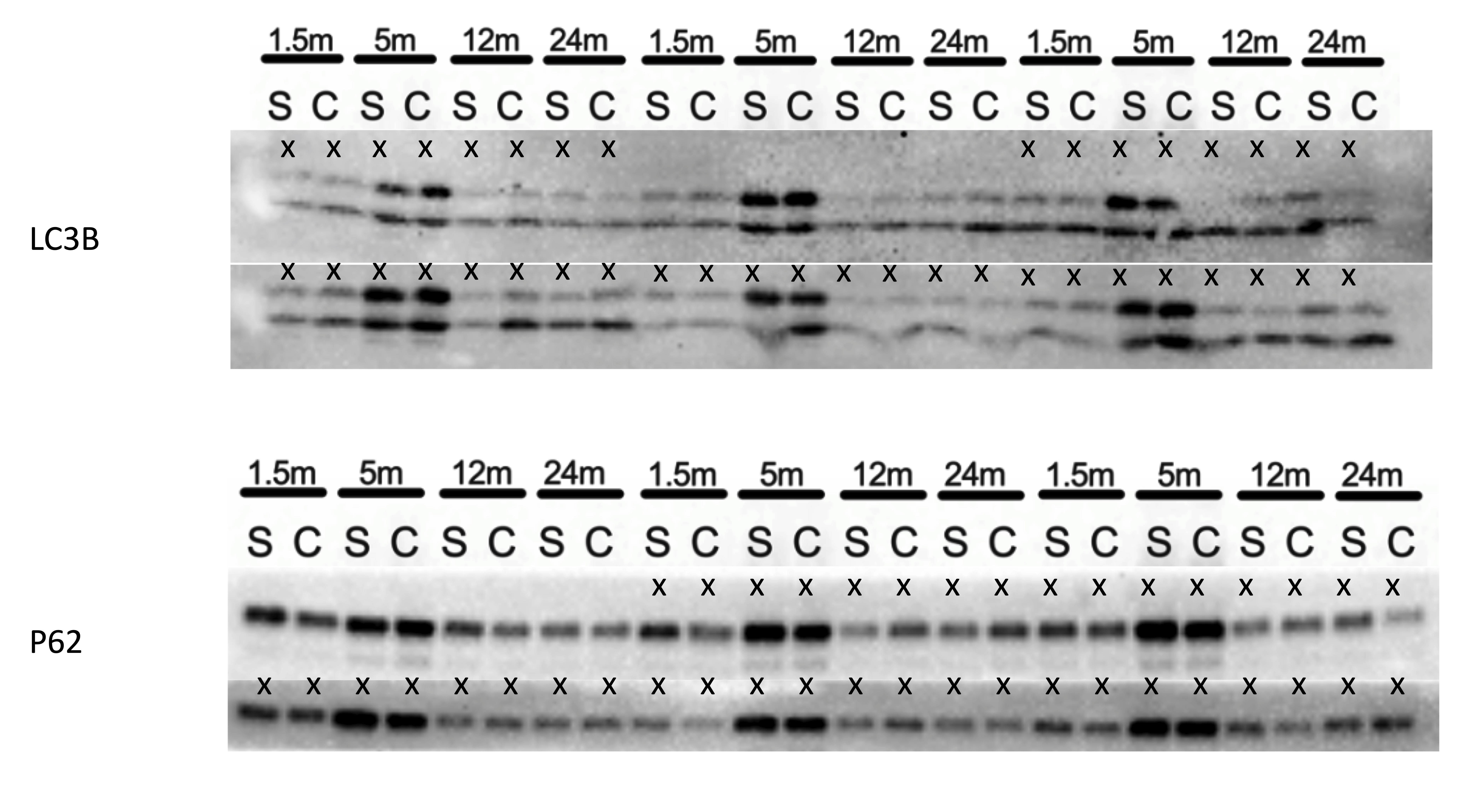

Supplement: S3 Fig — (TIFF) [file pone.0240278.s003.tiff]
